# Melatonin biosynthesis and regulation in reproduction

**DOI:** 10.3389/fendo.2025.1630164

**Published:** 2025-07-28

**Authors:** Jingjing Zhong, Zhiyong Lu, Zhi Zhou, Ning Ma, YeJuan Li, JiaJia Hu, Bangbei Wan, Weiying Lu

**Affiliations:** Reproductive Medical Center, Hainan Women and Children’s Medical Center, Haikou, China

**Keywords:** melatonin, biosynthesis, antioxidant, inflammatory response and assisted reproduction, MT1, MT2

## Abstract

Melatonin, a neuroendocrine hormone widely present in animals, is a derivative of tryptophan secreted by the pineal gland. This hormone regulates animal circadian rhythms and can affect reproductive performance in many ways; for example, melatonin levels change in response to sunshine duration changes, which can inhibit or promote reproductive performance. In juvenile animals, melatonin inhibits estrus, whereas in mature animals, it promotes estrus. Melatonin regulates animal reproductive activities mainly through the hypothalamus–pituitary–gonad axis and through membrane binding receptor (MT1 and MT2) interactions. It effectively removes cellular free radicals that have strong antioxidant effects and can directly act on the reproductive system and even early embryos by improving tissue and cell anti-inflammatory and antioxidant functions, improving animal reproductive performance. Although modern human fertility is no longer affected by seasonal reproduction, the relationship between melatonin and human reproduction remains unclear. Melatonin is important for improving mitochondrial function, reducing free radical damage, and inducing oocyte maturation, which can improve the fertilization rate, promote embryo development, and positively affect *in vitro* fertilization and embryo transfer. Here, we describe the biosynthesis and regulation of melatonin and its secretion, the physiological function of melatonin, and its effects on animal reproductive performance and assisted reproduction.

## 1 Introduction

Melatonin, a hormone produced by the pineal gland, has garnered significant attention owing to its role in reproductive system regulation (1). Melatonin’s influence spans various reproductive stages, including gamete production, embryo implantation, and fetal development (2). Importantly, melatonin exerts diverse regulatory effects on reproduction, mainly through binding with its receptors MT1 and MT2, which are G protein-coupled receptors (GPCRs) that play crucial roles in mediating melatonin signaling (3). These receptors are involved in multiple reproductive processes, such as gametogenesis, gamete quality, reproductive rhythm, endocrine function, and embryonic development (3). Understanding the intricate mechanisms of melatonin biosynthesis and its impact on reproductive processes is crucial for advancing our knowledge of its basic scientific and clinical applications (4). Here, we explore the complexities of melatonin biosynthesis and its regulation in reproduction, shedding light on the latest research findings and their potential implications for reproductive health (5). Neuroendocrine hormones are essential for complex communication between the nervous and endocrine systems and regulate various physiological processes, including reproduction (6). Melatonin, a neuroendocrine hormone, is a potent signaling molecule that coordinates reproductive events by controlling the release of reproductive hormones and interacting with other systems, such as the immune system, to optimize reproductive functions (7). Melatonin has become a research focus in reproductive science in recent years because of its diverse roles in regulating fertility (8).

The suprachiasmatic nucleus in the anterior hypothalamus governs the biological clock, maintains circadian rhythms, and synchronizes physiological processes with the natural light–dark cycle (9). Melatonin, also known as the “hormone of darkness,” plays a central role in this system. In response to darkness, the pineal gland synthesizes and releases melatonin, which reaches peak levels at night and decreases during the day (10). Melatonin also directly affects the reproductive system (11). Melatonin biosynthesis and regulation are important for understanding the complex interplay between melatonin and reproductive processes (12). This interaction has attracted increasing attention because of the diverse and pivotal roles of melatonin in regulating reproductive functions (13). Investigating the intricate processes involved in melatonin biosynthesis and their impact on reproduction can provide valuable insights into the complexities of fertility and potentially result in the development of novel therapeutic approaches addressing reproductive disorders and infertility (14). The multifaceted roles of melatonin in modulating circadian rhythms and reproductive functions have piqued the interest of scientists and clinicians, underscoring the importance of this research area (15). Previous studies have indicated that melatonin plays a role in regulating different reproductive functions (8). For example, it influences the secretion of gonadotropin-releasing hormones, which are crucial for the production of reproductive hormones in the hypothalamus (16). Moreover, melatonin has been linked to the control of follicle development, ovulation, and embryo implantation (17). Thus, the essential role of melatonin in the complex process of reproduction cannot be overlooked.

Melatonin regulates circadian rhythms and significantly influences various aspects of fertility, including the timing of ovulation, sperm production, and embryo implantation (18). Elucidating the mechanisms underlying melatonin biosynthesis and its regulation in reproduction will provide valuable insights into fertility processes and enable the development of innovative treatments for reproductive disorders and infertility (14). Increasing research into the multifaceted roles of melatonin in circadian rhythm and reproductive function regulation has highlighted the importance of scientific and clinical research on this neuroendocrine hormone (19). The diverse roles of melatonin in fertility regulation underscore the importance of further exploration (20), and the complex interplay between melatonin biosynthesis and regulation in reproduction has sparked widespread interest in its potential role in assisted reproductive technology (ART) (21). As the demand for ART increases, there is a growing need to explore the potential impact of melatonin on the success rate of these techniques (22). Investigating the role of melatonin in ART can potentially reveal novel strategies that can increase the efficacy of fertility treatments and improve outcomes for individuals and couples seeking reproductive assistance (23). Melatonin research can potentially revolutionize ART approaches and address evolving challenges in assisted reproduction (24). Furthermore, understanding the influence of melatonin on ART can help advance the personalized and targeted approaches that are increasingly emphasized in reproductive medicine (25).

The potential role of melatonin in ART has recently garnered significant interest. Advancements in ART procedures necessitate the optimization of fertility treatments to increase success rates (25). Melatonin is a promising candidate for enhancing ART outcomes due to its antioxidant nature and ability to modulate reproductive processes. Researchers have investigated the effects of melatonin supplementation in conjunction with ART to assess its potential to improve oocyte quality, enhance embryo development, and optimize the success of *in vitro* fertilization procedures (26). Furthermore, understanding the specific mechanisms by which melatonin regulates follicle development, ovulation, and embryo implantation in humans can provide valuable insights into reproductive outcomes (27).

Understanding the effects of melatonin on the reproductive system is essential for developing personalized treatment approaches for individuals facing fertility challenges (28). Understanding the influence of melatonin on reproductive function provides insight into potential mechanisms for tailoring fertility treatments according to individuals’ melatonin levels and circadian rhythms (29). In this review, we discuss interactions among melatonin, circadian rhythms, and reproductive processes with a specific focus on the potential of melatonin in the context of ART, providing valuable insights into these processes that could revolutionize reproductive science and contribute to the development of more effective and personalized fertility treatments (30).

In brief, we explore how melatonin biosynthesis and control affect human reproduction, particularly the timing of puberty, the menstrual cycle, follicle development, ovulation, and embryo implantation, seeking to fill the knowledge gap regarding melatonin’s involvement in human reproduction and its potential influence on ART (31). Furthermore, we discuss the regulation and management of melatonin in the aforementioned processes (32), including the impact of melatonin on menstrual cycle regularity and timing and the potential effects of melatonin supplementation on ART, including pregnancy rates and oxidative stress reduction.

We hypothesized that melatonin is crucial for the regulation and enhancement of human reproductive processes; specifically, we hypothesized that melatonin levels and their biosynthesis and regulation contribute to the timing of puberty, the menstrual cycle, follicle development, ovulation, and embryo implantation (33). We further hypothesized that melatonin supplementation could improve the success rate of ART by increasing the quality of oocytes and embryos, increasing pregnancy rates, reducing oxidative stress, and promoting overall reproductive health.

## 2 Melatonin synthesis and secretion

Various tissues and organs can secrete melatonin; however, only the retinal tissue of the pineal gland and eye exhibits periodic secretory activity. The biosynthesis of melatonin shows a circadian rhythm and seasonal characteristics associated with external light conditions. Functionally, melatonin can link changes in external light signals and multiple physiological activity rhythms (34), has a good protective effect on the nervous system, regulates circadian changes, and has good therapeutic effects on psychiatric diseases (35). In addition, melatonin has broad-spectrum antibacterial (36) and immunoregulatory functions (37).

Melatonin was originally isolated and identified from the pineal glands of animals (38). Research has shown that various tissues and organs, including the gut and ovarian follicles, can also synthesize melatonin (39) as autocrine and paracrine signals. Vertebrate gastrointestinal tryptophan is a precursor of melatonin biosynthesis. Tryptophan is excreted from the small intestine into the blood circulation and actively taken up by pineal cells, in which 5-hydroxytryptophan is formed by tryptophan hydroxylase. Aryl alkylamine-N-acetyltransferase (AANAT) converts tryptophan hydroxylase into N-acetylserotonin, and acetylserotonin O-methyltransferase (ASMT) catalyzes the final conversion to melatonin (40). AANAT is the major rate-limiting enzyme in this process, and its biological activity can regulate the melatonin synthesis rate. The binding regulatory/binding sequences in the AANAT gene encode a binding switch for cAMP operation, and cAMP-catalyzed protein kinase promotes the formation of a complex with the 14-3–3 protein. This AANAT/14-3–3 complex shields melatonin from dephosphorylation and AANAT proteolysis and reduces the K (m) of serotonin, enhancing melatonin production (41). Suofu et al. (42) further established that melatonin is synthesized in the mitochondrial matrix. Melatonin is quickly released into the blood and spinal fluid (43), binds to plasma proteins, and is distributed throughout most tissues (44).

Additionally, many studies have demonstrated that some bacteria and fungi can synthesize melatonin (45). Tilden et al. (46) investigated the influence of light conditions on the aerobic photosynthetic bacterium *Eryrobacter longusits*, demonstrating that it could synthesize and secrete melatonin under weak light conditions. However, the efficiency and concentration of melatonin synthesis in the bacterium’s natural state were low and not conducive to isolation, purification, and application. Advances in research on microbial genomes, genetic engineering technologies, and fermentation engineering have resulted in the use of genetically engineered bacteria to synthesize substances that are of high value or that are difficult to obtain (47). The use of engineered bacteria to ferment and transform substances has the advantages of convenience, speed, safety, efficiency, and cost reduction. *Escherichia coli* is the most commonly used host bacterium. Some *E. coli* plasmids can encode T7 lysozyme, express large quantities of target proteins, and inhibit the normal expression of host self-proteins (48). Researchers have used genetic engineering technology to transform *Xanthomonas* rapeseed carrying the phenylalanine 4-hydroxylase (P4H) gene, rice 3-O-methyltransferase (COMT) gene, and *Streptomyces* white toxin synthesis gene construction vector into *E. coli* and used protein engineering and metabolic engineering technology to successfully express melatonin in these bacteria (48). The differences in melatonin synthesis pathways between vertebrates and microorganisms are comprehensively detailed in **Table 1**.

**Table 1:** Comparison of melatonin synthesis in vertebrates and microorganisms.

Characteristics	Vertebrates	Microorganisms	References
Primary Source	Pineal gland, retina, other tissues	Specific bacteria (e.g., *Erythrobacter longus*), fungi	(40, 46)
Key Enzymes	TPH, AADC, AANAT, ASMT	Similar enzymes, but often with lower efficiency and concentration	(40, 46)
Regulation	Circadian rhythms, external light conditions	Light conditions (e.g., weak light for *Erythrobacter longus*)	(40, 46)
Production Method	Endogenous synthesis in specific tissues	Genetic engineering and fermentation processes (e.g., using *Escherichia coli*)	(46, 48)
Efficiency	High efficiency, regulated by complex biological processes	Lower efficiency in natural conditions but can be enhanced through genetic modifications	(46, 48)
Applications	Regulation of circadian rhythms, reproductive processes	Potential for industrial production and therapeutic applications	(46, 48)

TPH, tryptophan hydroxylase; AADC, aromatic L-amino acid decarboxylase; AANAT, arylalkylamine N-acetyltransferase; ASMT, acetylserotonin O-methyltransferase.

## 3 The biological function of melatonin

**Figure 1:**
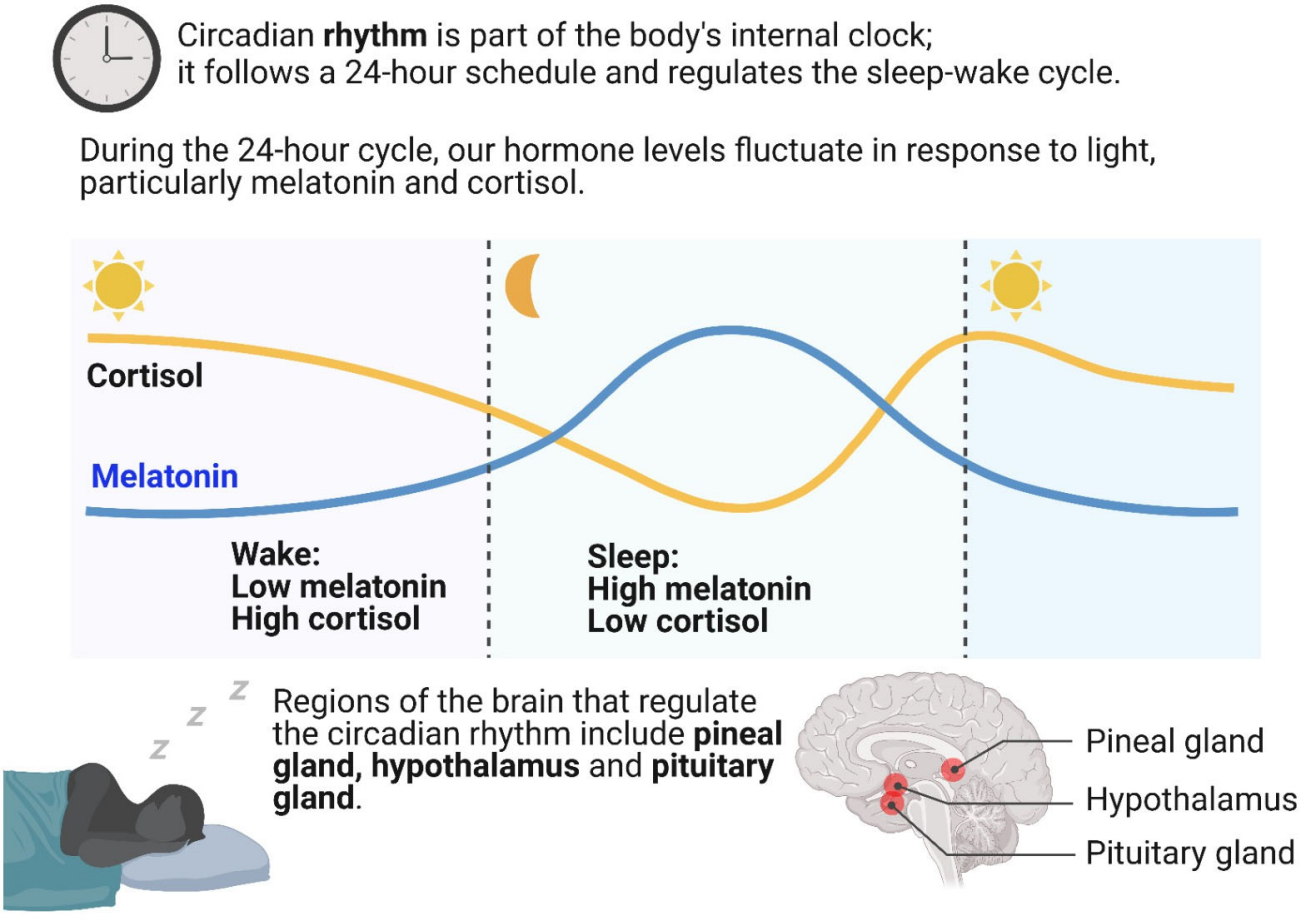
Biological function of melatonin in the regulation of circadian rhythms.

Melatonin regulates circadian rhythms in animals (49). At night, under weak light conditions, endogenous melatonin is synthesized to promote sleep and improve sleep quality. During the day, melatonin secretion by the pineal gland is inhibited by light stimulation (35). Studies have shown that changes in external light conditions can affect the level of melatonin synthesis and secretion by the pineal gland through ocular processes (IOP) and subsequently regulate the body’s sleep state (50). Thus, the body senses changes in external light through the eyes and transmits signals to the pineal gland, which converts these signals into melatonin production responses, regulating the body’s circadian rhythm. A mouse model with downregulated melatonin expression has been constructed via gene editing technology (51), revealing significantly longer sleep durations in these mice compared to those with upregulated melatonin expression. These results demonstrate the regulatory effect of melatonin on the circadian rhythm. **Figure 1** shows the biological function of melatonin in circadian rhythm regulation.

The immunomodulatory functions of melatonin can have proinflammatory and anti-inflammatory effects under different inflammatory conditions and can improve the body’s resistance and resilience to exogenous or endogenous antigens (52). These effects are partly mediated by the melatonin receptors MT1 and MT2, which can trigger various downstream signaling pathways. These receptors mainly interact with Gi (including Gαi2 and Gαi3) and Gq/11 proteins, leading to the inhibition of adenylate cyclase (AC) activity and cAMP production. Additionally, MT1 receptors can stimulate the Gβγ-dependent PI3K/Akt and PKC/ERK pathways and activate K+ channels (Kir3) and Ca2+ channels (Cav2.2). MT2 receptors can stimulate Gαi-dependent PKC/ERK signaling and decrease intracellular cGMP levels (3). These signaling pathways collectively contribute to the diverse biological functions of melatonin, including its immunomodulatory effects (3). Melatonin stimulates reactive oxygen species (ROS) production in monocytes, which can activate other immune cells (52), promoting immune system defenses. Furthermore, melatonin can act on MT1 and MT2 on the surface of human monocyte U937 cells, stimulating intracellular ROS production; however, the concentration of ROS produced does not cause oxidative stress-related damage (53, 54). Activated monocytes can differentiate into macrophages and synthesize chemokines and related inflammatory factors to perform their immunomodulatory functions. In other studies, melatonin has been found to act on MT1 on the cell membrane surface of immune cells, upregulating the expression of the interleukin 2 (IL-2) receptor and alleviating the inhibitory effects of prostaglandins on IL-2 (55). In contrast, melatonin inhibits severe inflammation; dextran sodium sulfate-induced neuroinflammation and liver injury in mice were relieved by exogenous melatonin administration, which increased short-chain fatty acid production (56). Melatonin has also been found to inhibit the overactivation of intestinal fonnesin X receptor fibroblast growth factor 15 (FXR-FGF 15) and apoptotic signal-regulated kinase 1 (ASK 1) in the liver, alleviating intestinal inflammation and hepatic metabolic disorders.

Melatonin has broad-spectrum antioxidant activity (37) and can limit oxidative damage through multiple mechanisms, including scavenging excess free radicals, stimulating endogenous antioxidant enzymes, and improving the efficiency of other antioxidants (57). Furthermore, melatonin can be transferred by hydrogen atoms (hydrogen atom transfer, HAT), proton-coupled electron transfer (proton-coupled electron transfer, PCET), free radical addition, substances (radical adduct formation, RAF), single-electron transfer (SET), sequential electron-proton transfer (SEPT), and other mechanisms that directly remove peroxy groups (peroxyl radical, ROO), hydroxyl radical (OH), OO groups (nitric oxide, NO), and other free radicals (58). Mekhloufi et al. (59) constructed an *in vitro* lipid model to assess the scavenging effects of melatonin and hydroperoxides. The results revealed that melatonin could directly react with and remove hydroxyl radicals. Melatonin can also chelate Cu^2+^, reduce Fe^2+^, Zn^2+^, Al^3+^, Mn^2+^, and other toxic metal ions (60), reduce metal ion-catalyzed molecular damage, and suppress metal ion interaction with β-amyloid peptides to produce free radicals (61), playing significant antioxidant and protective roles in the body. The effects of melatonin on Cu^2+^-mediated lipid peroxidation (62) and Cu^2+^/H_2_O_2_-induced metal-catalyzed oxidation and protein damage (63) have been found to be protective. In addition, melatonin can increase the antioxidant effects of glutaglyanin, ascorbic acid, and water-soluble vitamin E through electron transfer (64).

## 4 Effects of melatonin on reproduction

Melatonin can act on the hypothalamic–pituitary–gonadal axis to regulate animal reproductive activity (65), which has multiple effects on animal reproduction. These effects are largely mediated by the melatonin receptors MT1 and MT2. For example, in the male reproductive system, melatonin can inhibit the expression of key steroidogenic genes (such as p450scc, p450c17, and StAR) in Leydig cells via MT1 receptors, thereby reducing testosterone synthesis (3). In the female reproductive system, MT1 receptors are widely distributed in the ovary and are crucial for melatonin-regulated activities, such as delaying the decline in fertility in female animals (3). Moreover, melatonin can improve oocyte development and fertilization capacity via a receptor-mediated demethylation mechanism, including an increase in Tet1 gene expression and a decrease in Dnmt1 gene expression (3). In immature animals, melatonin can inhibit the secretion of endogenous gonadotropin-releasing hormone (GnRH) (66) to inhibit sexual maturation, spontaneous ovulation, and the estrous response (67). Melatonin also plays a role in mature animals (68). To some extent, this phenomenon shows that melatonin has a protective effect on immature animals, preventing premature reproductive activities and reducing damage to the reproductive system and other tissues and organs. The hypothalamic suprachiasmatic nucleus of mammals receives light stimulation through the retinal hypothalamic bundle, thereby regulating the rhythmicity of pineal melatonin synthesis and secretion. Melatonin is mainly secreted at night, and its secretion is inversely proportional to the duration of sunshine during the day. Thus, the photoperiod signal is transformed into changes in melatonin content in the animal, thereby regulating the reproductive cycle of animals with seasonal estrus (69, 70).

### 4.1 Effects on the male reproductive system

Melatonin significantly improves the status of the male reproductive system, acting through the hypothalamic–pituitary–gonadal (HPG) axis to regulate the secretion of reproductive hormones, primarily GnRH and luteinizing hormone (LH) (71). Melatonin receptors (MT1 and MT2) mediate not only melatonin signaling but also other hormone signaling, thereby increasing testosterone levels (72). In the embryonic and juvenile stages, melatonin promotes the secretion of male hormones and the development and maturation of the reproductive system by binding to MT1 and MT2 in testicular tissues (73). In adulthood, melatonin continues to promote testosterone secretion and sperm formation, increasing reproductive activity, especially in seasonal breeders (74).

The inherent anti-inflammatory and antioxidant effects of melatonin also contribute to the overall health of the male reproductive system. Studies have shown that melatonin can improve the survival of cryopreserved spermatogonial stem cells (SSCs) by reducing ROS production during freezing and thawing (75). Additionally, melatonin has a protective effect against reproductive diseases in male animals. For example, it can increase the phagocytic capacity of macrophages in the testis, inhibit the p38 MAPK pathway, and promote testosterone secretion by Leydig cells, thereby reducing inflammation (76).

### 4.2 Effects on the female reproductive system

Melatonin also significantly affects the female reproductive system, influencing oocyte maturation, cumulus cell expansion, and the production of steroid hormones. Recent studies have highlighted the critical role of melatonin in modulating gene expression levels related to these processes.

Melatonin has been shown to upregulate the expression of several genes crucial for oocyte maturation and cumulus cell expansion. For example, melatonin supplementation has been found to increase the expression of genes such as growth differentiation factor 9 (GDF9), bone morphogenetic protein 15 (BMP15), and pentraxin 3 (PTX3) (77). These genes are essential for promoting the development and quality of oocytes, thereby enhancing reproductive outcomes. Melatonin also supports the expansion of cumulus cells, which is vital for providing a supportive environment for oocyte maturation.

In addition to its effects on gene expression, melatonin influences the production of steroid hormones by granulosa cells. Growing granulosa cells produce steroid hormones such as 17β-estradiol and androstenedione, which are essential for maintaining the physical connections between granulosa cells and oocytes and facilitating the exchange of necessary substances during oocyte growth (78). Melatonin modulates the synthesis of these hormones, thereby supporting the overall health and development of oocytes.

The antioxidant and anti-inflammatory properties of melatonin contribute to its beneficial effects on the female reproductive system (79). By reducing oxidative stress and inflammation, melatonin helps protect oocytes and surrounding cells from damage, thereby improving the overall reproductive environment (80). This protective effect is particularly important in the context of ART, where oxidative stress can negatively impact oocyte quality and embryo development. The ability of melatonin to mitigate oxidative stress and inflammation ensures a healthier reproductive environment, thereby increasing ART procedure success rates.

The clinical implications of the effects of melatonin on the female reproductive system are significant. In the context of ART, melatonin supplementation has been shown to improve oocyte quality, increase embryo development, and increase pregnancy rates. For example, a systematic review and meta-analysis demonstrated that melatonin improved the fertilization rate and the number of mature oocytes (MII), although it did not significantly affect the clinical pregnancy rate (81). These findings highlight the potential of melatonin as a therapeutic agent for improving reproductive outcomes.

In conclusion, melatonin plays a vital role in modulating reproductive functions in both males and females and is a promising therapeutic agent for improving reproductive outcomes due to its antioxidant properties and effects on gene expression and steroid hormone production. Future research should continue to explore the mechanisms underlying the actions of melatonin and its potential applications in clinical settings.

### 4.3 Effects of melatonin on early embryonic development and oocyte quality

#### 4.3.1 Effects on early embryonic development

Melatonin affects both gamete formation and early embryo development. Melatonin can increase the mtRNA copy number, mitochondrial membrane potential, and mitochondrial distribution in blastocyst-stage cells; inhibit the expression of apoptotic genes such as p53 and Bax; and promote the expression of antioxidant genes such as SOD1 and GPx 4, thus improving the mitochondrial function of blastocyst-stage embryos, promoting early embryo development, and improving the quality of blastocysts (82). MT1 expression is initiated early in embryonic development (83), and MT1 is distributed mainly on the cell membrane in activated oocytes with no cleavage. With the development of blastomeres, MT1 receptor expression gradually increases, and it is primarily localized on the cell membrane. During embryo development, MT1 is expressed and distributed inside the blastomere, and its expression levels are low in degenerated embryos. Treating IVF-fertilized follicles with 10–9 mol/L melatonin significantly improved the blastocyst formation rate and embryo quality (84). During bovine embryo development, 10–9 mol/L melatonin significantly improved the cleavage rate, blastocyst rate, and number of blastocyst cells. Moreover, melatonin binds to MT2 receptors to promote the establishment of an endometrial receptive state during embryonic colonization (85). The expression of AANAT, the rate-limiting enzyme for melatonin synthesis, increases in the uterus during early pregnancy, and MT2 receptors are specifically expressed in uterine luminal epithelial cells and uterine glands on the second day of pregnancy (86). After the injection of 15 mg/kg melatonin, the endometrial thickness and uterine gland density increased, and the number of implantation sites and litter size increased significantly.

#### 4.3.2 Effects on oocyte quality during *in vitro* maturation and growth

Recent research has highlighted the significant role of melatonin in improving oocyte quality, particularly in the context of *in vitro* maturation and growth (87, 88). Melatonin has been shown to enhance the meiotic and developmental competence of oocytes derived from early antral follicles and small antral follicles (89). For example, studies have demonstrated that melatonin supplementation during *in vitro* maturation (IVM) can upregulate the expression of genes related to oocyte maturation, such as GDF9, BMP15, and PTX3 (77). These genes are essential for promoting oocyte development and improving oocyte quality. Besides, recent studies further demonstrate that melatonin supplementation during *in vitro* growth (IVG) of oocytes from preantral or early antral follicles significantly improves their developmental competence. For instance, a study has reported that melatonin could enhance the developmental potential of porcine oocyte-granulosa cell complexes derived from preantral follicles (90), while another research has showed that melatonin, combined with cyclic adenosine monophosphate (cAMP) modulators, could promote meiotic and developmental competence in porcine oocytes from early antral follicles during IVG and pre-maturation culture (91). These findings highlight melatonin’s dual role in supporting both oocyte growth and maturation, offering promising avenues for human ART applications.

Moreover, the antioxidant properties of melatonin play crucial roles in protecting oocytes from oxidative stress *in vitro* (92). Melatonin helps maintain oocyte integrity and functionality by scavenging ROS and enhancing endogenous antioxidant enzyme activity (57, 58). This protection is particularly important in IVM settings, where oocytes are exposed to relatively high levels of oxidative stress due to the absence of the natural protective mechanisms provided by the follicular environment.

Recent studies have also shown that melatonin can improve the survival and developmental competence of oocytes derived from small antral follicles (89). Compared with larger follicles, these oocytes often exhibit lower quality and developmental potential. Melatonin supplementation has been shown to enhance the meiotic maturation and developmental competence of these oocytes, thereby improving their potential for successful fertilization and embryo development (85).

In summary, the effects of melatonin on early embryonic development and oocyte quality are multifaceted. Melatonin enhances mitochondrial function, reduces oxidative stress, and promotes the expression of genes crucial for oocyte maturation and development. These findings underscore the potential of melatonin as a valuable supplement in IVM and *in vitro* growth (IVG) protocols, thereby improving reproductive outcomes.

## 5 Clinical application and treatment of melatonin in reproduction

ART has played a pivotal role in helping infertile couples achieve pregnancy. This includes a range of treatments, such as artificial insemination (AI), *in vitro* fertilization–embryo transfer (IVF–ET), and derivative technologies. The global ART-facilitated birth population has now exceeded 6 million, with ART treatment accounting for 1–3% of the total number of newborns in developed countries (93). One controlled clinical trial revealed that fertilization and pregnancy rates were approximately twice as high in patients treated with melatonin than in those treated without melatonin, indicating the ability of melatonin to improve the success rate of IVF–ET (94). A meta-analysis of randomized trials revealed that melatonin treatment significantly increased clinical pregnancy rates during the ART cycle, as did the number of oocytes collected, mature oocytes, and high-quality embryos (95). Melatonin can also improve the clinical outcomes of IVF–ET by increasing the fertilization rate and the number of mature oocytes and high-quality embryos (96); therefore, melatonin intake during IVF–ET is considered to have some clinical utility (97).

Melatonin is also used as a therapeutic agent for unexplained infertility. A randomized pilot study revealed improvements in intracellular oxidative stress and oocyte quality in patients receiving 3 or 6 mg/day doses and slight increases in clinical pregnancy and live birth rates with IVF–ET (98). The main advantage of melatonin antioxidant therapy is its relatively adequate safety, as confirmed in short-term studies. However, long-term clinical trials are needed to evaluate its application further.

Despite these promising findings, the effectiveness of melatonin supplementation in ART can vary significantly depending on the specific stage of treatment and the individual context. One key limitation is the variability in individual responses to melatonin treatment. Factors such as age, underlying health conditions, and the presence of other antioxidants in the body can significantly impact the efficacy of melatonin. For example, older patients or those with preexisting oxidative stress conditions may not respond as positively to melatonin treatment as younger or healthier individuals do. This variability underscores the need for personalized approaches in ART, where melatonin supplementation is tailored to the specific needs and conditions of each patient.

Another limitation is the lack of standardized protocols for melatonin administration in ART. The optimal dosage, timing, and duration of melatonin supplementation remain the foci of ongoing research. The variability in these parameters across different studies makes it challenging to draw definitive conclusions about the universal effectiveness of melatonin in ART. Standardizing these protocols is crucial for maximizing the benefits of melatonin while minimizing potential side effects.

Furthermore, while melatonin has shown promise in improving oocyte quality and embryo development, its impact on the overall success rates of ART, such as live birth rates, has been less consistent. Some studies have demonstrated significant improvements in live birth rates with melatonin supplementation, whereas others have not reported such effects (99–102). This variability highlights the need for further research to better understand the contexts in which melatonin is most effective. Future studies should focus on elucidating the mechanisms underlying individual variability in response to melatonin treatment, potentially leading to personalized approaches in ART.

**Table 2** encapsulates the multifaceted role of melatonin in reproductive medicine, summarizing its beneficial effects on oocyte quality, embryo development, fertilization rates, clinical pregnancy rates, and live birth rates. These findings also underscore the antioxidant and anti-inflammatory properties of melatonin, highlighting the need for further research to elucidate optimal dosing strategies and long-term clinical outcomes.

**Table 2:** Summary of the effects of melatonin on reproduction.

Characteristics	Effect of melatonin	Relevant references
Oocyte Quality	Enhances oocyte maturation and quality by upregulating genes such as GDF9, BMP15, and PTX3.	(77, 85)
Embryo Development	Increases mitochondrial function and reduces apoptosis in blastocysts.	(82, 84)
Fertilization Rates	Significantly increases fertilization rates in IVF-ET procedures.	(94, 95)
Clinical Pregnancy Rates	Increases clinical pregnancy rates during ART cycles.	(94–96)
Live Birth Rates	Shows potential for increasing live birth rates, although results vary.	(98)
Antioxidant Effects	Reduces oxidative stress in oocytes and embryos.	(57, 58, 96)
Anti-inflammatory Effects	Mitigates inflammation in reproductive tissues.	(59, 60)

GDF9, growth differentiation factor 9; BMP15, bone morphogenetic protein 15; PTX3, pentraxin 3; IVF-ET, *in vitro* fertilization-embryo transfer; ART, assisted reproductive technology.

## 6 Conclusion

Melatonin is a key signaling molecule that connects changes in external light conditions with changes in physiological activities in the body. After the retina of an animal’s eye receives a light signal, it transmits the signal to the pineal gland. The pineal gland then transforms the light signal into melatonin, thereby participating in various physiological reactions in the body. Melatonin regulation of various antioxidants and immune responses improves animal reproductive performance. ART has enhanced our understanding of the various physiological changes in the reproductive process, and an increasing number of problems restricting reproductive performance have been identified. Previous studies have elucidated the role of melatonin in improving animal reproductive performance, and this factor can potentially improve human-assisted reproduction in the future. However, the mode of administration of melatonin and its isoforms requires further study in different species and breeds.
